# Supplementary material for: Sequential phosphorylation of NDEL1 by the DYRK2-GSK3β complex is critical for neuronal morphogenesis
Source: eLife. 2019 Dec 9;8:e50850. doi: 10.7554/eLife.50850 (PMC6927744; doi:10.7554/eLife.50850)
Supplement: Supplementary file 1. [file elife-50850-supp1.docx]

| **Key Resources Table** | | | | |
| --- | --- | --- | --- | --- |
| **Reagent type (species) or resource** | **Designation** | **Source or reference** | **Identifiers** | **Additional information** |
| Genetic reagent (*M. musculus*) | IcrTac:ICR | IMSR | Cat# TAC:icr, RRID:IMSR_TAC:icr |  |
| Genetic reagent (*R. norvegicus*) | NTac:SD | RGD | Cat# 1566440, RRID:RGD_156644 |  |
| Cell line (*H. sapiens*) | HEK293 | ATCC | Cat# PTA-4488, RRID:CVCL_0045 |  |
| Cell line (*H. sapiens*) | SH-SY5Y | ATCC | Cat# CRL-2266, RRID:CVCL_0019 |  |
| Cell line (*M. musculus*) | NIH3T3 | ATCC | Cat# CRL-6442, RRID:CVCL_0594 |  |
| Antibody | Anti-NDEL1 (Rabbit polyclonal) | Proteintech Group | Cat# 17262-1-AP, RRID:AB_2235821 | WB (1:1000)  IP (1:600)  ICC (1:200)  IHC (1:200) |
| Antibody | Anti-pNDEL1 (pS332/336) (Rabbit polyclonal) | This paper |  | WB (1:1000)  ICC (1:100)  IHC (1:100)  Materials and Methods – Antibodies and plasmids |
| Antibody | Anti-TARA (Rabbit polyclonal) | Thermo Fisher Scientific | Cat# PA5-29092, RRID:AB_2546568 | WB (1:1000)  IP (1:1000)  ICC (1:200) |
| Antibody | Anti-DYRK2 (599542) (Mouse monoclonal) | Thermo Fisher Scientific | Cat# MA5-24269, RRID:AB_2606267 | WB (1:500) |
| Antibody | Anti-GSK-3beta (3D10) (Mouse monoclonal) | Cell Signaling Technology | Cat# 9832, RRID:AB_10839406 | WB (1:1000)  IP (1:600) |
| Antibody | Anti-FLAG (Rabbit polyclonal) | Sigma-Aldrich | Cat# F7425, RRID:AB_439687 | WB (1:2000)  ICC (1:200) |
| Antibody | Anti-FLAG (M2) (Mouse monoclonal) | Sigma-Aldrich | Cat# F1804, RRID:AB_262044 | WB (1:2000)  IP (1:1000)  ICC (1:200) |
| Antibody | Anti-GFP (Rabbit polyclonal) | Molecular Probes | Cat# A-11122, RRID:AB_221569 | WB (1:3000) |
| Antibody | Anti-GFP (B-2) (Mouse monoclonal) | Santa Cruz Biotechnology | Cat# sc-9996, RRID:AB_627695 | WB (1:1000)  IP (1:200) |
| Antibody | Anti-α-tubulin (DM1A) (Mouse monoclonal) | Santa Cruz Biotechnology | Cat# sc-32293, RRID:AB_628412 | WB (1:1000) |
| Antibody | Anti-α-tubulin (Mouse monoclonal) | Proteintech Group | Cat# 66031-1-Ig, RRID:AB_11042766 | WB (1:2000)  ICC (1:200) |
| Antibody | Anti-c-Myc (Mouse monoclonal) | Santa Cruz Biotechnology | Cat# sc-40, RRID:AB_627268 | WB (1:1000) |
| Antibody | Anti-actin (Goat polyclonal) | Santa Cruz Biotechnology | Cat# sc-1616, RRID:AB_630836 | WB (1:500) |
| Antibody | PhosphoSerine Antibody Q5 | Qiagen | Cat# 37430 | WB (1:500) |
| Antibody | Normal rabbit IgG (Isotype control) | Santa Cruz Biotechnology | Cat# sc-2027, RRID:AB_737197 |  |
| Antibody | Rabbit IgG, polyclonal – Isotype Control | Abcam | Cat# ab37415, RRID:AB_2631996 |  |
| Antibody | Normal mouse IgG (Isotype control) | Santa Cruz Biotechnology | Cat# sc-2025, RRID:AB_737182 |  |
| Antibody | Mouse IgG, HRP-linked whole Ab | GE Healthcare | Cat# NA931, RRID:AB_772210 | WB (1:7500) |
| Antibody | Rabbit IgG, HRP-linked whole Ab | GE Healthcare | Cat# NA934, RRID:AB_772206 | WB (1:7500) |
| Antibody | VeriBlot for IP Detection Reagent (HRP) | Abcam | Cat# ab131366 | WB (1:500) |
| Antibody | Goat Anti-Mouse IgG (H+L) Antibody, Alexa Fluor 568 Conjugated | Molecular Probes | Cat# A-11004, RRID:AB_141371 | ICC (1:200)  IHC (1:100) |
| Antibody | Goat Anti-Mouse IgG (H+L) Antibody, Alexa Fluor 647 Conjugated | Molecular Probes | Cat# A-21235, RRID:AB_141693 | ICC (1:200)  IHC (1:100) |
| Antibody | Goat Anti-Rabbit IgG (H+L) Antibody, Alexa Fluor 488 Conjugated | Molecular Probes | Cat# A-11008, RRID:AB_143165 | ICC (1:200)  IHC (1:100) |
| Antibody | Goat Anti-Rabbit IgG (H+L) Antibody, Alexa Fluor 568 Conjugated | Molecular Probes | Cat# A-11011, RRID:AB_143157 | ICC (1:200)  IHC (1:100) |
| Antibody | Goat anti-rabbit IgG, Flamma® 648 | BioActs | Cat# RSA1261 | ICC (1:200)  IHC (1:100) |
| Recombinant DNA reagent | CCSB-Broad Human Kinase ORF Collection | Johannessen et al., 2010 (PMID: 21107320); Yang et al., 2011 (PMID: 21706014) | Cat# Addgene_1000000014 |  |
| Recombinant DNA reagent | pFLAG-CMV2 | Sigma-Aldrich | Cat# E7033 |  |
| Recombinant DNA reagent | pEGFP-C3 | Clontech | Cat# 6082-1 |  |
| Recombinant DNA reagent | pcDNA3.1/myc-His | Invitrogen | Cat# V80020 |  |
| Recombinant DNA reagent | pCIG2-EGFP | Choe et al., 2013 (PMID: 23595735) |  |  |
| Recombinant DNA reagent | pCIG2-mRFP | Choe et al., 2013 (PMID: 23595735) |  |  |
| Recombinant DNA reagent | pEZYmyc-His | Guo et al., 2008 (PMID: 18064608) | RRID:Addgene_18701 |  |
| Recombinant DNA reagent | pEZYflag | Guo et al., 2008 (PMID: 18064608) | RRID:Addgene_18700 |  |
| Recombinant DNA reagent | FLAG-hNDEL1 | Hong et al., 2016 (PMID: 27546710) |  |  |
| Recombinant DNA reagent | FLAG-hNDEL1 ^S331A^ | This paper |  | Site-directed mutagenesis – Ser331 (TCC) > Ala331 (GCC) |
| Recombinant DNA reagent | FLAG-hNDEL1 ^S332A^ | This paper |  | Site-directed mutagenesis – Ser332 (TCG) > Ala332 (GCG) |
| Recombinant DNA reagent | FLAG-hNDEL1 ^S335A^ | This paper |  | Site-directed mutagenesis – Ser335 (TCG) > Ala335 (GCG) |
| Recombinant DNA reagent | FLAG-hNDEL1 ^S336A^ | This paper |  | Site-directed mutagenesis – Ser336 (TCA) > Ala336 (GCA) |
| Recombinant DNA reagent | FLAG-hNDEL1 ^S332/336A^ | This paper |  | Site-directed mutagenesis – Ser332 (TCG) > Ala332 (GCG) and Ser336 (TCA) > Ala336 (GCA) |
| Recombinant DNA reagent | pLL3.7-scrambled shRNA-EGFP | Hong et al., 2016 (PMID: 27546710) |  |  |
| Recombinant DNA reagent | pLL3.7-NDEL1 shRNA-EGFP | Hong et al., 2016 (PMID: 27546710) |  |  |
| Recombinant DNA reagent | pLL3.7-NDEL1 shRNA-EGFP NDEL1 ^resist1+WT^ | This paper |  | Insertion of CDS of shRNA-resistant NDEL1^WT^ after CMV promoter followed by EGFP |
| Recombinant DNA reagent | pLL3.7-NDEL1 shRNA-EGFP NDEL1 ^resist+S332/336A^ | This paper |  | Insertion of CDS of shRNA-resistant NDEL1^S332/336A^ after CMV promoter followed by EGFP |
| Recombinant DNA reagent | pCIG2-mRFP-NDEL1 ^resist+WT^ | This paper |  | Subcloned from shRNA resist FLAG-NDEL1^resi^ - Hong et al., 2016 (PMID: 27546710) |
| Recombinant DNA reagent | pCIG2-mRFP-NDEL1 ^resist+S332/336A^ | This paper |  | Site-directed mutagenesis – Ser332 (TCG) > Ala332 (GCG) and Ser336 (TCA) > Ala336 (GCA) |
| Recombinant DNA reagent | GFP-hTARA | Hong et al., 2016 (PMID: 27546710) |  |  |
| Recombinant DNA reagent | GFP-hTARA ^Δ413-499^ | Hong et al., 2016 (PMID: 27546710) |  |  |
| Recombinant DNA reagent | MYC-hTARA | Hong et al., 2016 (PMID: 27546710) |  |  |
| Recombinant DNA reagent | FLAG-hTARA | Hong et al., 2016 (PMID: 27546710) |  |  |
| Recombinant DNA reagent | pCIG2-EGFP-hTARA | This paper |  | Subcloned from GFP-hTARA |
| Recombinant DNA reagent | pCIG2-EGFP-hTARA ^Δ413-499^ | This paper |  | Subcloned from GFP-hTARA ^Δ413-499^ |
| Recombinant DNA reagent | pCIG2-EGFP-mTARA | This paper |  | Subcloned from Cath.A differentiated (CAD) cell cDNA library |
| Recombinant DNA reagent | pCIG2-EGFP-mTARA ^Δ401-487^ | This paper |  | Subcloned from pCIG2-EGFP-mTARA with fusion-PCR method |
| Recombinant DNA reagent | GFP-hGSK3β^S9A^ | This paper |  | Subcloned from HEK293 cell cDNA library followed by site-directed mutagenesis – Ser9 (TCC) > Ala9 (GCC) |
| Recombinant DNA reagent | FLAG-hGSK3β | This paper |  | Subcloned from HEK293 cell cDNA library |
| Recombinant DNA reagent | FLAG-hDYRK2 | This paper |  | Subcloned from HEK293 cell cDNA library |
| Recombinant DNA reagent | GFP-hDYRK2 | This paper |  | Subcloned from HEK293 cell cDNA library |
| Recombinant DNA reagent | pCIG2-EGFP hGSK3β^S9A^-FLAG hDYRK2 | This paper |  | Subcloned from GFP-hGSK3β^S9A^ and FLAG-hDYRK2 |
| Recombinant DNA reagent | RFP-N1-LifeAct | This paper |  | Materials and Methods - Time-lapse live imaging with FRAP assay |
| Recombinant DNA reagent | mCherry-α-tubulin | This paper | RRID:Addgene_49149 |  |
| Recombinant DNA reagent | pUBC-NDEL1 ^resist+WT^-IRES-mRFP | This paper |  | Subcloned from pCIG2-mRFP-NDEL1 ^resist+WT^ |
| Recombinant DNA reagent | pUBC-NDEL1 ^resist+S332/336A^-IRES-mRFP | This paper |  | Subcloned from pCIG2-mRFP-NDEL1 ^resist+S332/336A^ |
| Recombinant DNA reagent | GFP-hLIS1 | Hong et al., 2016 (PMID: 27546710) |  |  |
| Recombinant DNA reagent | GFP-hDYNC1I1 | This paper |  | Subcloned from HEK293 cell cDNA library |
| Recombinant DNA reagent | pCIG2-mRFP-mNde1 | This paper |  | Subcloned from Cath.A differentiated (CAD) cell cDNA library |
| Recombinant DNA reagent | hCas9_D10A | Mali et al., 2013 (PMID: 23287722) | RRID:Addgene_41816 |  |
| Recombinant DNA reagent | mNdel1-exon9-gRNA#1 | This paper |  | Core sequence: TCTTC TCGCC GTAGT GCCGT |
| Recombinant DNA reagent | mNdel1-exon9-gRNA#2 | This paper |  | Core sequence: ATTGA TATCG CGCAG AGTCC |
| Recombinant DNA reagent | KnockIn-Donor-WT-EGFP | This paper |  | Subcloned from mouse genomic DNA |
| Recombinant DNA reagent | KnockIn-Donor-S332/336A-EGFP | This paper |  | Subcloned from mouse genomic DNA |
| Recombinant DNA reagent | pHAGE-UBC-GFP-LAMP1 | Lee et al., 2017 (PMID: 28223337) |  |  |
| Commercial assay or kit | LR Clonase™ II Plus enzyme | Invitrogen | Cat# 123538 |  |
| Commercial assay or kit | MAX Efficiency™ DH5α™ Competent Cells | Invitrogen | Cat# 18258012 |  |
| Commercial assay or kit | Pierce C18 Spin Columns | Themo Fisher Scientific | Cat# 89870 |  |
| Chemical compound, drug | Alexa Fluor 568 Phalloidin | Thermo Fisher Scientific | Cat# A12380 |  |
| Chemical compound, drug | Alkaline Phosphatase, Calf Intestinal (CIP) | New England Biolabs | Cat# M0290 |  |
| Chemical compound, drug | Clarity Western ECL Substrate | Bio-Rad | Cat# 1705061 |  |
| Chemical compound, drug | cOmplete Protease Inhibitor Cocktail | Roche | Cat# 11697498001 |  |
| Chemical compound, drug | Ketamine hydrochloride | Yuhan Corporation |  |  |
| Chemical compound, drug | Laminin | Corning | Cat# 354239 |  |
| Chemical compound, drug | Lithium chloride solution (8 M, for molecular biology, ≥99%) | Sigma-Aldrich | Cat# L7026 |  |
| Chemical compound, drug | Lipofectamine 2000 | Invitrogen | Cat# 11668019 |  |
| Chemical compound, drug | Poly-D-lysine hydrobromide | Sigma-Aldrich | Cat# P6407 |  |
| Chemical compound, drug | UltraCruz® Aqueous Mounting Medium with DAPI | Santa Cruz Biotechnology | Cat# sc-24941, RRID:AB_10189288 |  |
| Chemical compound, drug | Vivamagic | Vivagen | Cat# VM001 |  |
| Chemical compound, drug | Xylazine | Bayer AG | N/A |  |
| Software, algorithm | ImageJ (Fiji) | Schindelin et al., 2012 (PMID: 22743772) | RRID:SCR_002285 |  |
| Software, algorithm | Imaris | Bitplane | RRID:SCR_007370 |  |
| Software, algorithm | Olympus cellSens Software | Olympus | RRID:SCR_016238 |  |
| Software, algorithm | easyFRAP-web | Koulouras et al., 2018 (PMID: 29901776) | N/A |  |
| Software, algorithm | GraphPad Prism | GraphPad | RRID:SCR_002798 |  |
| Software, algorithm | Proteome Discoverer | Thermo Fisher Scientific | RRID:SCR_014477 |  |
| Other | Olympus Confocal Laser Scanning Microscope Fluoview FV3000 | Olympus | RRID:SCR_017015 |  |
| Other | Q-Exactive Plus Orbitrap | Thermo Fisher Scientific |  |  |
